# Dietary Immunostimulant CpG Modulates MicroRNA Biomarkers Associated with Immune Responses in Atlantic Salmon (*Salmo salar*)

**DOI:** 10.3390/cells8121592

**Published:** 2019-12-07

**Authors:** Xi Xue, Nardos Tesfaye Woldemariam, Albert Caballero-Solares, Navaneethaiyer Umasuthan, Mark D. Fast, Richard G. Taylor, Matthew L. Rise, Rune Andreassen

**Affiliations:** 1 Department of Ocean Sciences, Memorial University of Newfoundland, St. John’s, NL A1C 5S7, Canada; acaballeroso@mun.ca (A.C.-S.); navaumasuthan@gmail.com (N.U.); 2 Department of Life Sciences and Health, Faculty of Health Sciences, OsloMet–Oslo Metropolitan University, N-0130 Oslo, Norway; nate@oslomet.no (N.T.W.); rune.andreassen@oslomet.no (R.A.); 3 Hoplite Laboratory, Department of Pathology and Microbiology, Atlantic Veterinary College, University of Prince Edward Island, Charlottetown, PE C1A 4P3, Canada; mfast@upei.ca; 4 Cargill Animal Nutrition, 10383 165th Avenue NW, Elk River, MN 55330, USA; richard_taylor@cargill.com

**Keywords:** CpG ODN, immunostimulant, functional feed, immune response, miRNA, biomarker, sequencing, Atlantic salmon

## Abstract

MicroRNAs (miRNAs) are key regulators in fish immune responses. However, no study has previously characterized the impact of polyriboinosinic polyribocytidylic acid (pIC) and formalin-killed typical *Aeromonas salmonicida* (ASAL) on miRNA expression in Atlantic salmon fed a commercial diet with and without immunostimulant CpG. To this end, first, we performed small RNA deep sequencing and qPCR analyses to identify and confirm pIC- and/or ASAL-responsive miRNAs in the head kidney of salmon fed a control diet. DESeq2 analyses identified 12 and 18 miRNAs differentially expressed in pIC and ASAL groups, respectively, compared to the controls. Fifteen of these miRNAs were studied by qPCR; nine remained significant by qPCR. Five miRNAs (miR-27d-1-2-5p, miR-29b-2-5p, miR-146a-5p, miR-146a-1-2-3p, miR-221-5p) were shown by qPCR to be significantly induced by both pIC and ASAL. Second, the effect of CpG-containing functional feed on miRNA expression was investigated by qPCR. In pre-injection samples, 6 of 15 miRNAs (e.g., miR-181a-5-3p, miR-462a-3p, miR-722-3p) had significantly lower expression in fish fed CpG diet than control diet. In contrast, several miRNAs (e.g., miR-146a-1-2-3p, miR-192a-5p, miR-194a-5p) in the PBS- and ASAL-injected groups had significantly higher expression in CpG-fed fish. Multivariate statistical analyses confirmed that the CpG diet had a greater impact on miRNA expression in ASAL-injected compared with pIC-injected fish. This study identified immune-relevant miRNA biomarkers that will be valuable in the development of diets to combat infectious diseases of salmon.

## 1. Introduction

Worldwide demand for seafood for human consumption, including a growing contribution from aquaculture (~50% in 2016), continues to climb because of a flat or decreasing global wild fisheries production in the face of rising human population [1,2,3]. Consequently, there is great potential for the aquaculture industry to expand. With a variety of species being farmed, Atlantic salmon (*Salmo salar*) is one of the most economically important species in aquaculture [4]. Infectious diseases have resulted in substantial mortality and losses to Atlantic salmon aquaculture worldwide, affecting the growth and sustainability of the industry [5]. Several well-known viruses that cause severe diseases in Atlantic salmon are RNA viruses [6]. These include viruses with single-stranded RNA genomes (e.g., salmonid alphavirus (SAV), infectious salmon anemia virus (ISAV) and viral hemorrhagic septicemia virus (VHSV)) and double-stranded RNA genomes (e.g., infectious pancreatic necrosis virus (IPNV)) [6]. Bacterial pathogens that have a severe impact on salmonid aquaculture include *Piscirickettsia salmonis* (which causes piscirickettsiosis or salmonid rickettsial septicaemia) [7], *Aeromonas salmonicida* (the cause of furunculosis) [8], *Renibacterium salmoninarum* (the cause of bacterial kidney disease) [9], and *Moritella viscosa* (the cause of winter ulcer disease) [10].

Microbial cell components (e.g., lipopolysaccharide, peptidoglycan, RNAs, and DNAs), recognized by animal immune cells as pathogen-associated molecular patterns (PAMPs), can elicit host immune responses to fight the invading pathogen [11]. The detection of PAMPs by specific pattern-recognition receptors (PRRs) on or within the host immune cells triggers intracellular signaling cascades that increase the expression of soluble mediators (e.g., both pro-inflammatory and anti-inflammatory cytokines), which can lead to increased phagocytosis, bactericidal activity, respiratory burst, antiviral and complement activities [12]. Taking advantage of this mechanism, researchers have used polyriboinosinic polyribocytidylic acid (pIC), a synthetic double-stranded RNA (dsRNA), to elicit antiviral responses [5,13,14,15], and formalin-killed *Aeromonas salmonicida* (ASAL), a bacterin, to elicit antibacterial responses [16,17].

Immune response-mediated gene expression can be regulated through small non-coding RNAs (ncRNAs) including microRNAs (miRNAs) [18,19,20,21]. miRNAs are important regulators of gene expression at the post-transcriptional level [18,22]. The primary miRNA transcripts (pri-miRNAs) are cleaved by Drosha into shorter miRNA precursors (pre-miRNAs). Thereafter, pre-miRNAs are exported out of the nucleus and further processed by Dicer to produce two small mature miRNAs (i.e., 5p and 3p) that are usually 20–24 nt in length [22]. Typically, one of the mature miRNAs is then assembled into the miRNA-induced silencing complex (miRISC), which can exercise its gene-silencing function by binding mainly to the 3′ untranslated region (UTR) of target mRNA [20].

Recent advances in high-throughput sequencing technology (e.g., small RNA deep sequencing) and bioinformatics tools have led to the detection of virus/bacteria-responsive miRNAs in teleosts [19,21,23,24]. For instance, twenty differentially expressed miRNAs were identified in Atlantic salmon challenged with SAV; the majority of the predicted mRNA targets were involved in promoting the inflammatory response [19]. Analyses of Atlantic salmon tissues infected with *P. salmonis* revealed 84 and 25 differentially expressed miRNAs in head kidney and spleen, respectively; functional annotation of predicted mRNA targets of *P. salmonis*-responsive miRNAs showed involvement in the immune response, such as genes related to chemokine-mediated signaling pathway and neutrophil chemotaxis [23]. Such studies have improved our understanding of miRNAs involved in immune responses in teleosts [18]. However, the impact of pIC-triggered antiviral and ASAL-triggered antibacterial immune responses on the miRNA expression in Atlantic salmon were previously uncharacterized.

Over recent decades the development of aquafeeds has continued to progress with diets being more specifically designed to meet the nutritional needs of species, stage of the life cycle, and health status of the fish [25]. Functional feeds are diets designed to have positive effects on both the health and growth of the animals ingesting them by supplying additional functional ingredients beyond the basic nutritional requirements of the animal [26]. Components that act as immunostimulants are often added to the feeds, and can generally increase resistance to disease by enhancing the non-specific immune system [25,26]. For example, algal and plant extracts as dietary immunostimulants have been tested in different fish species; other dietary supplements containing PAMPs as immunostimulants also showed promising results in mitigating fish diseases [11].

A type of PAMP commonly used as an immunostimulant is unmethylated DNA, which contains cytosine–phosphate–guanine oligodeoxynucleotide motifs (CpG ODN) [27]. Bacterial genomes, some viral genomes and invertebrate genomes differ structurally from vertebrate genomes, which exhibit CpG suppression and methylation [27,28]. Unmethylated DNA, containing CpG motifs, acts as a danger signal to the vertebrate host and triggers an immune response [27]. The immune response induced by CpG is mediated through Toll-like receptor 9 (TLR9), a PRR present on the cell surface or within endosomal compartments of B cells, dendritic cells, and macrophages [29,30]. Based on the backbone structure and oligonucleotide sequences, synthetic CpG ODNs are divided into three classes (i.e., A-, B-, and C-classes) with distinct immunomodulating properties [28]. B-class CpG ODNs primarily stimulate the proliferation of B cells [31]. For example, CpG ODN 205 (i.e., B-class) stimulated the immune system of turbot (*Scophthalmus maximus*), and induced protection against bacterial challenge [30]. Another B-class ODN, CpG ODN 1668, was shown to activate immune responses against iridovirus infection in rock bream (*Oplegnathus fasciatus*) [32], and *Vibrio parahaemolitycus* challenge in Pacific red snapper (*Lutjanus peru*) [31]. In addition, protection against sea lice (*Lepeophtheirus salmonis*) infection in Atlantic salmon by orally administered CpG ODN 1668 (10–20 mg kg^−1^ feed) has been reported [27,33]. Nevertheless, the impact of dietary CpG on the expression of miRNAs associated with antiviral and antibacterial responses in fish including Atlantic salmon was previously unknown.

In the present study, we investigated the host miRNA expression responses to viral mimic pIC and bacterin ASAL stimulations in the head kidney of Atlantic salmon fed a control diet by a deep sequencing approach. Head kidney was chosen as the target tissue as it plays an important role in the specific and non-specific defense mechanisms in teleost fish, and its role in hematopoiesis is equivalent to bone marrow in higher vertebrates [4,34,35]. Putative antiviral and antibacterial responsive miRNAs identified through sequencing were also studied by qPCR in fish fed a functional feed (control diet top-coated with CpG ODN 1668). The expression of these candidate miRNAs was measured before and 24 h after PAMP injections. This study allowed us to identify miRNAs that are valuable biomarkers for responses to pIC and ASAL stimulations in the head kidney of Atlantic salmon, and to study the influence of this CpG-containing functional feed on the expression of immune-relevant miRNAs.

## 2. Materials and Methods

### 2.1. Feed Production

EWOS Dynamic S feed (5 mm; 27% fat, 46% protein) was used in this experiment as the control diet and base feed for the functional diet (referred as CpG diet). The CpG diet was produced by dissolving CpG ODN 1668 (Integrated DNA Technologies, Coralville, IA, USA) components in distilled water and spraying onto the pellets. Then, coated pellets were brought under −0.9 bar of vacuum for 10 min, followed by a drying step at 60 °C for 30 min to remove excess water, to obtain a final concentration of 10 mg kg^−1^ of feed. The CpG coating procedures were carried out at the Chute Animal Nutrition Centre of Dalhousie University Agricultural Campus (Truro, NS, Canada).

### 2.2. Feeding Trial, Immune Challenge, and Fish Sampling

**Figure 1:**
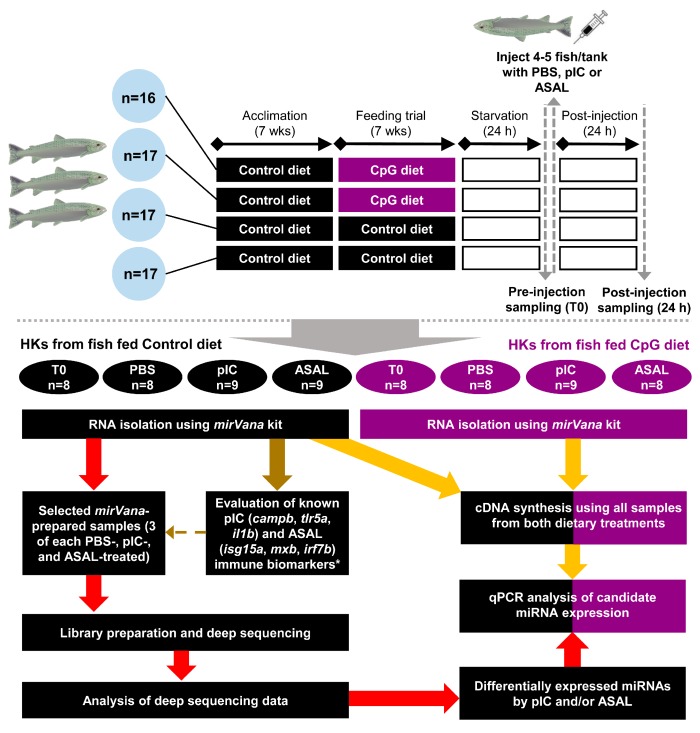
Overview of experimental design. Following 7 weeks of feeding trial, fish fed both diets were subjected to immune challenge by an intraperitoneal (IP) injection of sterile phosphate-buffered saline (PBS), bacterial antigen *Aeromonas salmonicida* (ASAL), or viral mimic polyriboinosinic polyribocytidylic acid (pIC). *mirVana*-prepared head kidney (HK) templates from three of each PBS-, pIC-, and ASAL-injected fish (control diet only) were selected for deep sequencing based on the qPCR assessed expression of known pIC (i.e., *isg15a*, *mxb*, *irf7b*) and ASAL (i.e., *campb*, *tlr5a*, *il1b*) immune biomarker transcripts. Selected pIC- and/or ASAL-responsive miRNAs were studied by qPCR using all samples from both dietary groups. *mRNA qPCR analyses were conducted using DNase-treated and column-purified total RNA.

The Atlantic salmon feeding trial was conducted at the Dr. Joe Brown Aquatic Research Building (JBARB, Ocean Sciences Centre (OSC), Memorial University of Newfoundland, St. John’s, NL, Canada). Salmon smolts were obtained from Northern Harvest Sea Farms (Stephenville, NL, Canada), transported to the JBARB and held in 3800 L tanks. After arrival, salmon were PIT (passive integrated transponder)-tagged and fed with the control diet before the start of the feeding trial. Atlantic salmon (post-smolts; 232 ± 52 g mean initial weight ± SD; *n* = 67) were randomly distributed among four 620 L tanks (16–17 fish per tank). After 7 weeks of acclimation, salmon from 2 tanks were switched from the control diet to the CpG diet while the other two tanks remained on the control diet for another 7 weeks. Fish were kept in a flow-through seawater system (~10–11 °C, dissolved oxygen ≥ 10 mg L^−1^) under a 24 h light photoperiod. Fish were fed to apparent satiation using automatic feeders (AVF6 Vibratory Feeder; Pentair Aquatic Eco-Systems, Inc., Nanaimo, BC, Canada), which were set to vibrate for 3 s hourly from 5 pm to 3 am. The daily ration was set at 1% of the average body weight (BW) of the salmon in each tank, which was estimated using their initial weight (for each tank, individually) and assuming an exponential growth of 1% BW/day. Satiation was assessed by monitoring the amount of uneaten pellets the next morning. An overview of the experimental design, including the feeding trial, immune challenges and subsequent molecular analyses (discussed below), is illustrated in Figure 1.

At the end of the feeding trial, both dietary groups were subjected to immune challenge by an intraperitoneal (IP) injection (25 gauge needle) of bacterial antigen ASAL or viral mimic pIC. Fish were starved for 24 h, after which 4 fish per tank (8 per treatment) were euthanized with an overdose of MS-222 (400 mg L^−1^, Syndel Laboratories, Vancouver, BC, Canada) and dissected for time 0 (i.e., pre-injection) head kidney samples. Formalin-killed typical ASAL was obtained in the form of a vaccine (Furogen Dip, Elanco (formerly Novartis), Charlottetown, PE, Canada). The ASAL solution was prepared as in Hori et al. [17], while the pIC (Catalogue # P0913; Sigma-Aldrich, Oakville, ON, Canada) was diluted in sterile phosphate-buffered saline (PBS; Gibco, Carlsbad, CA, USA) at 2 µg µL^−1^ for injection. Then, 4–5 salmon per tank (i.e., 8–9 per treatment) were lightly anesthetized (50 mg L^−1^ of MS-222) and injected with 1 µL of pIC, ASAL or PBS per g of wet mass. Fish were then sampled 24 h post-injection as described above. Body weight, fork length, and liver weight of fish were measured. Head kidney samples (50–100 mg) were collected, flash-frozen in liquid nitrogen, and stored at −80 °C until RNA extraction. This study was carried out in accordance with the animal care protocol 17-77-MR, approved by the Institutional Animal Care Committee of Memorial University of Newfoundland.

### 2.3. RNA Isolation

Total RNAs of all collected head kidney samples were extracted using the *mirVana* miRNA isolation kit (Ambion/Life Technologies, Carlsbad, CA, USA) according to the manufacturer’s instructions. The RNA integrity was verified by 1% agarose gel electrophoresis, and RNA purity was assessed by A260/280 and A260/230 using NanoDrop spectrophotometry (Thermo Fisher, Mississauga, ON, Canada). All RNA samples used in this study showed tight 18S and 28S ribosomal RNA bands and A260/230 ratios greater than 2. Also, A260/280 ratios of most of the samples were higher than 1.9; 3 out of 67 samples had A260/280 ratios between 1.7 and 1.9.

### 2.4. Library Preparation and Deep Sequencing

Prior to the selection of the samples for deep sequencing, aliquots of *mirVana*-prepared total RNAs from all fish fed control diet were subjected to DNase treatment and column purification, as described in Caballero-Solares et al. [36]. These RNAs were subjected to qPCR analyses of known ASAL- (i.e., cathelicidin antimicrobial peptide b (*campb*), *tlr5a*, interleukin-1 beta (*il1b*)) (Caballero-Solares et al. manuscript in preparation) and pIC- (i.e., interferon stimulated gene 15a (*isg15a*), interferon-induced GTP-binding protein b (alias myxovirus resistance b, *mxb*), interferon regulatory factor 7b (*irf7b*)) [5] responsive immune biomarker transcripts, to ensure the efficacy of the immune challenges and to select representative individuals for deep sequencing. The qPCR analyses of these immune biomarkers were conducted as described in Caballero-Solares et al. [36]. Details on the methods and results for immune biomarker mRNA qPCR are provided in Supplemental Table S1. The *mirVana*-prepared total RNAs from three of each PBS-, ASAL-, and pIC-injected individuals fed control diet were selected for miRNA sequencing analyses (see Supplemental Table S1 for qPCR-based sample selection). Small RNA library construction and sequencing were performed at the Norwegian Genomics Consortium (NGC)’s Genomics Core Facility. All sequencing libraries were generated using the NEBNext Small RNA Library Prep Set for Illumina (New England Biolabs, Ipswich, MA, USA) with 1 µg of total RNA input, following the manufacturers’ instructions. In brief, *mirVana*-prepared total RNAs were ligated with 3′ and 5′ RNA adapters, followed by reverse transcription (RT) and PCR enrichment using barcoded RT-primers. The resulting cDNA products were purified using 6% polyacrylamide gels, and size selection of fragments (approximately 145–160 bp) was carried out to enrich small RNAs. The sequencing was performed on a NextSeq 500 instrument (Illumina, Inc, San Diego, CA, USA), producing 75 bp single-end reads.

### 2.5. Analysis of Deep Sequencing Data

The quality of raw sequencing reads (fastq files) was assessed using FastQC toolkit (v.0.11.5; http://www.bioinformatics.babraham.ac.uk/projects/fastqc), to ensure that the quality was satisfactory before adaptor sequences were removed using cutadapt (v.1.13) [37]. The trimmed sequence reads were size-filtered to discard reads that were outside the expected size range of mature miRNAs (18–25 nt). The quality of the trimmed and size-filtered reads was checked by a second FastQC analysis. All deep sequencing reads have been submitted to the NCBI Sequence Read Archive (SRA) database (BioProject PRJNA555179).

The clean sequence reads were aligned to a reference index consisting of all known mature miRNAs in Atlantic salmon [22], using STAR aligner software (v.2.5.2b) [38]. The alignment files (BAM format) were further processed in R using the *featureCounts* function from the *Rsubread* package to produce count matrices [39]. These count tables were used as input to test for differential expression of miRNAs using the R package DESeq2 [40]. Differentially expressed miRNAs were identified by comparing the ASAL or pIC groups to the PBS group (control) (*n* = 3 from each experimental condition). miRNAs were considered to be differentially expressed if they had Benjamini-Hochberg adjusted *p*-value of ≤ 0.10.

### 2.6. Prediction of Target Genes and Their Functional Annotations

The miRNA target prediction tool RNAhybrid [41] was applied to identify the putative target genes of the pIC- and/or ASAL-responsive miRNAs identified by the DESeq2 analyses. The mature miRNA sequences were tested against 3′UTRs from all Atlantic salmon transcripts in the NCBI Reference Sequence database (Refseq; https://www.ncbi.nlm.nih.gov/refseq/). The following parameters were applied in the RNA hybrid analysis: helix constraint 2–8, no G:U in seed, and a minimum free energy threshold of −18 kcal/mol. Gene ontology (GO) terms of the predicted target genes from Atlantic salmon were obtained from UniProt Knowledgebase (http://www.uniprot.org/). Based on the GO term annotations and published studies, a subset of predicted target genes with functions associated with immune response were identified. Cross-reference links from the UniProt database were further used to retrieve organism-specific pathway annotations from the online resource Kyoto Encyclopedia of Genes and Genomes (KEGG) pathway database (https://www.genome.jp/kegg/pathway.html).

### 2.7. qPCR Analysis of miRNA Expression

The expression of 15 miRNAs (5 pIC-responsive, 7 ASAL-responsive, 3 commonly responsive to both pIC and ASAL) (see Supplemental Table S2 for qPCR primers), selected from the DESeq2 analyses, was quantified by qPCR using samples from all individuals (i.e., 8–9 samples per treatment). In addition to fish fed control diet, the qPCR experiment also included head kidney samples from fish fed CpG containing diet and subjected to the immune stimulations.

cDNA templates for qPCR were synthesized in 20-μL reactions from 400 ng of *mirVana* extracted total RNA using miScript II RT Kit (Qiagen, Hilden, Germany) as recommended by the manufacturer’s instructions. The cDNAs were diluted by adding 180 μL of RNase-free water (Qiagen) for use in the qPCR assays. PCR amplifications were performed in duplicate using 12.5 μL of 2× QuantiTect SYBR Green PCR Master Mix, 2.5 μL of 10× miScript Universal Primer, 2.5 μL specific forward primer (10 μM), 5 μL RNase-free water (Qiagen), and 2.5 μL of diluted cDNA template representing 5 ng of input total RNA. All qPCR assays were conducted in an AriaMx Real-time PCR System (Agilent Technologies, Santa Clara, CA, USA) using 96-well plates. The real-time analysis program consisted of 1 cycle of 95 °C for 15 min, and 40 cycles of 94 °C for 15 s, 55 °C for 30 s and 70 °C for 30 s, followed by a final melting point analysis.

All forward primers were designed based on the mature sequences of miRNAs of interest (Table 1), while a universal primer, provided by the miScript SYBR Green PCR Kit (Qiagen), was used as a reverse primer in each qPCR assay. Quality testing ensured that a single product was amplified (dissociation curve analysis) and that there was no primer-dimer present in the no-template control except for miR-181a-5-3p. Amplification efficiencies [42] were calculated using cDNA synthesized from head kidney RNA samples (*n* = 6; 2 of each PBS-, ASAL-, and pIC-injected) that had been pooled post-cDNA synthesis. Standard curves were generated using a 4-5-point 1:3 dilution series. Two miRNAs (miR-25-3p and miR-17-5p), suggested as the most suitable normalizers for miRNA expression in Atlantic salmon [43], were used as normalizers in the current study. These normalizers were expressed stably in our qPCR study (i.e., the geometric mean of normalizers’ C_T_ less than 0.3 cycles different for injection-matched groups or diet-matched groups) (see Supplemental Table S3 for normalizer C_T_ values). Agilent AriaMx software v1.5 was applied to obtain C_T_ (or Cq) values. The relative quantity (RQ) of each miRNA was determined using a qBase relative quantification framework [44,45], with normalization to both miR-25-3p and miR-17-5p, and with amplification efficiencies incorporated. For pre-injection samples (i.e., T0 samples), the RQs of each miRNA were calibrated against fish fed the control diet, while for IP-injected groups, the RQs of each miRNA were calibrated against PBS-injected fish fed the control diet.

### 2.8. Statistical Analyses

All qPCR data (i.e., RQs) were subjected to Grubbs’ test to identify potential outliers and then log_2_-transformation to meet the normality assumption. In total, 19 RQ values were identified as statistical outliers in the entire dataset (i.e., out of 765 RQ values), and excluded from the study. Each miRNA of interest had a minimum of 7 samples per treatment. For pre-injection samples, miRNA expression differences between diet groups were determined using a Student’s *t*-test (*p* < 0.05). For IP-injected groups, miRNA expression differences between treatments and diets were determined using two-way analysis of variance (ANOVA), followed by a Dunnett’s test to assess the effect of PAMPs within each dietary group (i.e., pIC/ASAL vs. PBS), and a Student’s t-test to assess the dietary effect within treatment groups (*p* < 0.05). All of the statistical tests above were performed in Prism v7.0 (GraphPad Software Inc., La Jolla, CA, USA).

Principal coordinates analysis (PCoA), permutational multivariate ANOVA (PERMANOVA), and similarity of percentages analysis (SIMPER) were performed using PRIMER (Version 6.1.15; PRIMER-E Ltd, Ivybridge, UK) to explore the differences in qPCR-analyzed miRNA expression among samples from fish fed different diets (control vs. CpG) and in different treatment groups (pre-injection, PBS-, pIC- and ASAL-injected).

## 3. Results

### 3.1. Deep Sequencing and Identification of Differentially Expressed miRNAs

A deep sequencing approach was used to discover pIC- or ASAL-responsive miRNAs in the head kidney of salmon fed the control diet. Table 1 provides an overview of the read numbers obtained from the deep sequencing of the samples used in the present study. The total number of raw reads obtained from sequencing for all samples ranged from 9.6 to 34.5 million. After trimming and filtering, the number of clean reads for all samples ranged from 4.8 to 10.0 million reads. More than 68% of clean reads (i.e., after trimming and size filtering) were mapped to a recent update of Atlantic salmon reference miRNAome (i.e., all known mature miRNAs) [22]. The sequencing results of all samples are available in the SRA database of NCBI under the BioProject PRJNA555179 (Table 1).

DESeq2 analyses (adjusted *p*-value < 0.10) were applied to identify miRNAs that were pIC- or ASAL-responsive in the head kidney of Atlantic salmon. This revealed 12 mature miRNAs that were significantly upregulated in the pIC group when compared to the PBS-injected control group; the expression of these miRNAs was 1.6- to 14.0-fold higher in the pIC group (Table 2). Only one miRNA (miR-106a-3p) showed decreased expression (−1.9-fold) in the pIC group (Table 2). The comparison of the ASAL group against the PBS-injected control group revealed 16 significantly up-regulated miRNAs; the expression of these miRNAs was 1.5- to 17.2-fold higher in the ASAL group (Table 3). Two miRNAs (miR-722-3p and miR-727a-3p) had decreased expression (−2.1-fold and −1.8-fold) in the ASAL group (Table 3). In addition, 3 miRNAs (miR-146a-1-2-3p, miR-221-5p, miR-146-5p) were upregulated in both pIC and ASAL groups compared with the PBS controls (Table 2 and Table 3). The mature sequences and miRBase identities of all pIC- and/or ASAL-responsive miRNAs are given in Supplemental Table S4.

The predicted target genes of the pIC- and/or ASAL-responsive miRNAs from the DESeq2 analysis were identified by in silico analysis against the 3′UTRs from the Atlantic salmon transcriptome (i.e., mRNA Refseq database). A total of 1591 genes were identified as putative targets of pIC- and/or ASAL-responsive miRNAs (Supplemental Table S5). The gene ontology annotations of these genes (retrieved from the UniProt database) revealed 130 of them have immune-relevant functions (Supplemental Tables S6 and S7). Within these 130 immune-relevant predicted target genes, 24 and 54 were unique targets associated with pIC- and ASAL-responsive miRNAs, respectively; 52 target genes were in common (Supplemental Tables S6 and S7). Among the immune-relevant predicted targets of pIC-responsive miRNAs, 27 could be mapped to species-specific KEGG pathways; while 35 could be mapped for predicted targets of ASAL-responsive miRNAs. These KEGG pathways included NOD-like receptor signaling pathway, cytokine–cytokine receptor interaction, necroptosis, Toll-like receptor signaling pathway, apoptosis, C-type lectin receptor signaling pathway, RIG-I-like receptor signaling pathway, and cell adhesion molecules (CAMs) (Table 4). The two KEGG pathways that had the most target genes assigned in both putative target gene lists were NOD-like receptor signaling pathway and cytokine–cytokine receptor interaction (Table 4).

### 3.2. qPCR Validation of DESeq2-Identified pIC- and/or ASAL-Responsive miRNAs

Fifteen miRNAs (5 pIC-responsive, 7 ASAL-responsive, three commonly responsive to both pIC and ASAL) identified as differentially expressed by DESeq2 were successfully subjected to qPCR analyses to confirm the sequencing results using larger numbers of biological replicates (*n* = 8–9) than were included in the sequencing study (Table 2 and Table 3). qPCR analyses on miRNA expression of fish fed the control diet are discussed below. Fold-change values and significance are summarized in Table 2 and Table 3. Among deep sequencing-identified pIC-responsive miRNAs, the results from qPCR analyses agreed well (i.e., statistically significant) with the results from DESeq2 analyses for 7 of the 8 miRNAs tested; while not statistically significant, qPCR for miR-135bd-5p revealed the same direction of change as shown by deep sequencing (Table 2). Among the 10 deep sequencing-identified ASAL-responsive miRNAs subjected to qPCR analyses, 5 of these (50%), namely miR-29b-2-5p, miR-146a-5p, miR-146a-1-2-3p, miR-221-5p and miR-727a-3p, were confirmed (i.e., statistically significant) by qPCR; 4 of the remaining miRNAs showed the same direction of change (i.e., up- or down-regulation) as the sequencing results (Table 3).

**Figure 2:**
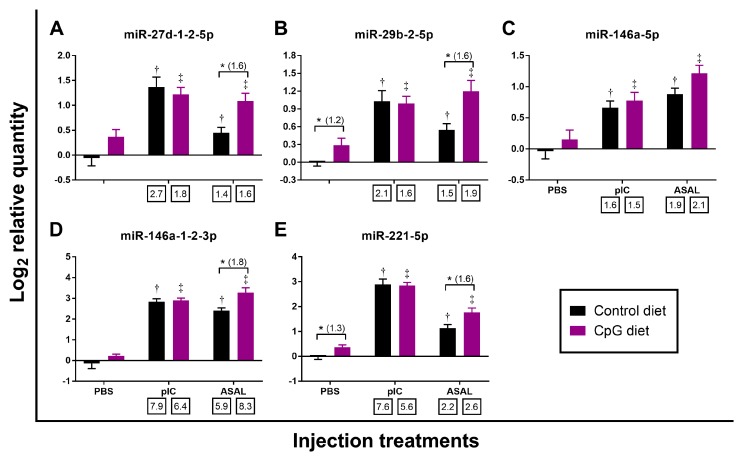
qPCR analyses of miRNAs identified by deep sequencing as being responsive to both pIC and ASAL injections (*n* = 8–9). Average log_2_ RQs with SE bars are plotted. An asterisk (*) represents a significant difference between diets in each injection treatment (*p* < 0.05) with fold-change given in brackets. A dagger (†) or diesis (‡) represents a significant difference between PAMP-injected salmon and the diet-matched PBS-injected control (*p* < 0.05) with fold-change indicated below the *x*-axis. For qPCR fold-change calculation, overall fold up-regulation was calculated as 2^A−B^ as in Xue et al. [46], where A is the mean of log_2_ RQ from the pIC or ASAL groups, and B is the mean of log_2_ RQ from the diet-matched PBS group. (A) miR-27d-1-2-5p; (B) miR-29b-2-5p; (C) miR-146a-5p; (D) miR-146a-1-2-3p; (E) miR-221-5p.

**Figure 3:**
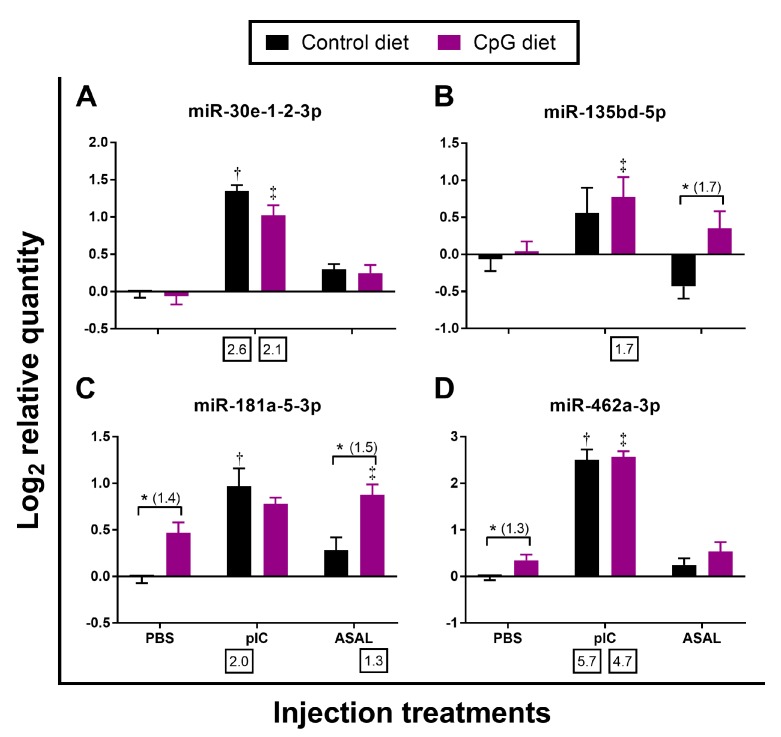
qPCR analyses of miRNAs identified by deep sequencing as being responsive to pIC alone (*n* = 8–9). Average log_2_ RQs with SE bars are plotted. An asterisk (*) represents a significant difference between diets in each injection treatment (*p* < 0.05) with fold-change given in brackets. A dagger (†) or diesis (‡) represents a significant difference between the pIC/ASAL-injected salmon and the diet-matched PBS-injected control (*p* < 0.05) with fold-change indicated below the *x*-axis. For qPCR fold-change calculation, overall fold up-regulation was calculated as 2^A−B^ as in Xue et al. [46], where A is the mean of log_2_ RQ from the pIC or ASAL groups, and B is the mean of log_2_ RQ from the diet-matched PBS group. (**A**) miR-30e-1-2-3p; (**B**) miR-135bd-5p; (**C**) miR-181a-5-3p; (**D**) miR-462a-3p.

**Figure 4:**
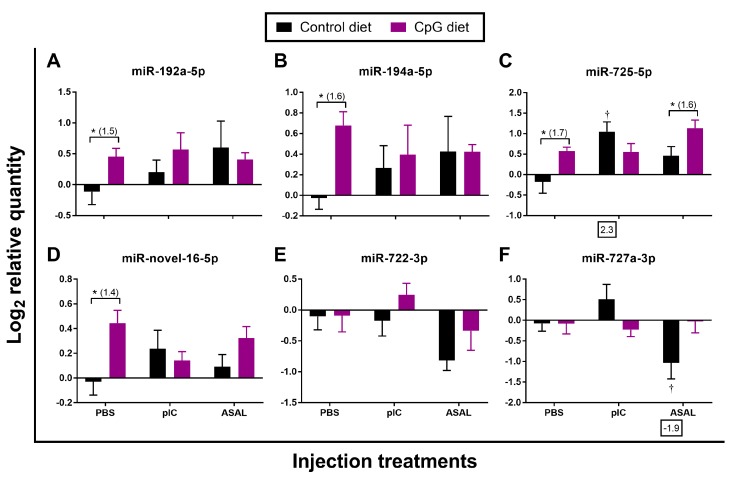
qPCR analyses of miRNAs identified by deep sequencing as being responsive to ASAL alone (*n* = 8–9). Average log_2_ RQs with SE bars are plotted. An asterisk (*) represents a significant difference between diets in each injection treatment (*p* < 0.05) with fold-change given in brackets. A dagger (†) represents a significant difference between PAMP-injected salmon and the diet-matched PBS-injected control (*p* < 0.05) with fold-change indicated below the *x*-axis. For qPCR fold-change calculation, overall fold up-regulation was calculated as 2^A−B^ as in Xue et al. [46], where A is the mean of log_2_ RQ from the pIC or ASAL groups, and B is the mean of log_2_ RQ from the diet-matched PBS group. For down-regulated miRNAs, fold-change values were inverted (−1/fold-change). (**A**) miR-192a-5p; (**B**) miR-194a-5p; (**C**) miR-725-5p; (**D**) miR-novel-16-5p; (**E**) miR-722-3p; (**F**) miR-727a-3p.

In addition to 3 DESeq2-identified miRNAs (miR-146a-1-2-3p, miR-146a-5p, miR-221-5p) that were commonly responsive to both pIC and ASAL stimulations, the qPCR results also showed that miR-27d-1-2-5p and miR-29b-2-5p were significantly up-regulated by both stimulations when compared with the PBS-injected salmon fed the control diet (Table 2 and Table 3; Figure 2A,B). Among these 5 miRNAs, the expression of miR-146a-1-2-3p and miR-221-5p was more strongly induced by pIC stimulation (up to 7.9-fold) than miR-27d-1-2-5p, miR-29b-2-5p, and miR-146a-5p (up to 2.7-fold) (Table 2 and Table 3; Figure 2). The ASAL induction of miR-146a-1-2-3p (5.9-fold) was stronger than that of miR-27d-1-2-5p, miR-29b-2-5p, miR-146a-5p, and miR-221-5p (~2-fold) (Table 2 and Table 3; Figure 2). Among the miRNAs that were only responsive to pIC stimulation, the induction of miR-462a-3p (5.7-fold) was higher than miR-30e-1-2-3p (2.7-fold) and miR-181a-5-3p (2.2-fold) (Table 2; Figure 3A,C,D). For deep sequencing-identified miRNAs that were only responsive to ASAL, miR-727a-3p was shown by qPCR to be significantly down-regulated in ASAL-injected salmon compared with PBS control (Table 3; Figure 4F). It is worth noting that miR-725-5p was significantly up-regulated (2.3-fold) by pIC stimulation in fish fed the control diet (Figure 4C).

### 3.3. Impact of Diets on the Expression of pIC- and/or ASAL-Responsive miRNAs

**Figure 5:**
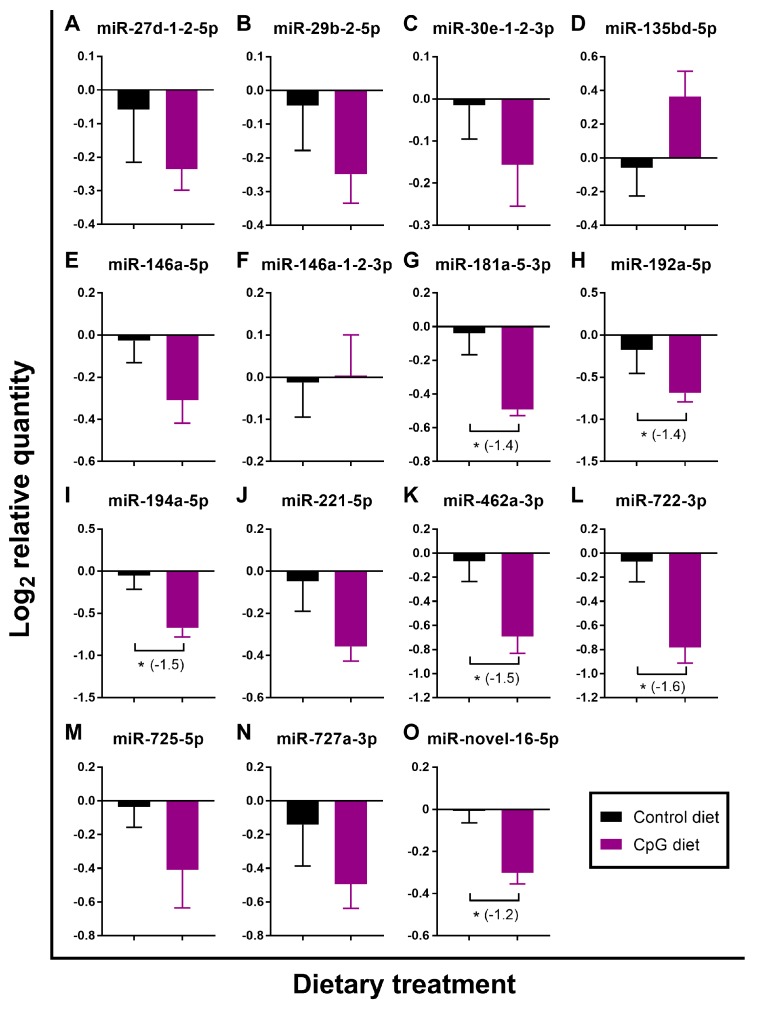
qPCR analyses of basal expression (pre-injection samples) of candidate pIC- and/or ASAL-responsive miRNAs identified by deep sequencing (*n* = 8). Average log_2_ RQs with SE bars are plotted. An asterisk (*) indicates a significant difference between diets for a given miRNA (*p* < 0.05) with fold-change given in brackets. For qPCR fold-change calculation, overall fold up-regulation was calculated as 2^A−B^ as in Xue et al. [46], where A is the mean of log_2_ RQ from the CpG group, and B is the mean of log_2_ RQ from the control group. For down-regulated miRNAs, fold-change values were inverted (−1/fold-change). (**A**) miR-27d-1-2-5p; (**B**) miR-29b-2-5p; (**C**) miR-30e-1-2-3p; (**D**) miR-135bd-5p; (**E**) miR-146a-5p; (**F**) miR-146a-1-2-3p; (**G**) miR-181a-5-3p; (**H**) miR-192a-5p; (**I**) miR-194a-5p; (**J**) miR-221-5p; (**K**) miR-462a-3p; (**L**) miR-722-3p; (**M**) miR-725-5p; (**N**) miR-727a-3p; (**O**) miR-novel-16-5p.

The putative pro-immune impact of the diet containing functional ingredient CpG ODN 1668 vs. the control feed was investigated in pre-injection head kidney samples (i.e., basal expression) by analyzing the gene expression of the 15 pIC- and/or ASAL-responsive miRNAs. This comparison revealed that 6 out of these miRNAs (i.e., miR-181a-5-3p, miR-192a-5p, miR-194a-5p, miR-462a-3p, miR722-3p, and miR-novel-16-5p) showed significant down-regulation by the CpG diet (−1.4, −1.4, −1.5, −1.5, −1.6, and −1.2-fold, respectively; Figure 5G–I,K,L,O). The remaining miRNAs assayed by qPCR except miR-135bd-5p and miR-146a-1-2-3p showed trends of lower expression in salmon fed the CpG diet compared to the controls (Figure 5).

In contrast to the pre-injection samples, several miRNAs in the PBS- and ASAL-injected groups, had higher expression in fish fed CpG diet compared to the controls (Figure 2, Figure 3 and Figure 4). In PBS-treated salmon, fish fed CpG diet showed significantly higher expression of the 8 miRNAs: miR-29b-2-5p, miR-221-5p, miR-181a-5-3p, miR-462a-3p, miR-192a-5p, miR-194a-5p, miR-725-5p, and miR-novel-16-5p (1.2-, 1.3-, 1.4-, 1.3-, 1.5-, 1.6-, 1.7-, and 1.4-fold, respectively) than those fed the control diet (Figure 2B,E, Figure 3C,D and Figure 4A–D). In ASAL-treated salmon, 7 miRNAs, namely miR-27d-1-2-5p, miR-29b-2-5p, miR-146a-1-2-3p, miR-221-5p, miR-135bd-5p, miR-181a-5-3p, and miR-725-5p, had significantly higher expression (1.6-, 1.6-, 1.8-, 1.6-, 1.7-, 1.5- and 1.6-fold, respectively) in fish fed the CpG diet compared to the fish fed the control diet (Figure 2A,B,D,E; Figure 3B,C; Figure 4C). Given the effect of the CpG diet in PBS and ASAL injected groups, it was an unexpected finding that no miRNA was significantly modulated by the CpG diet when compared to the control diet in the pIC-treated salmon (Figure 2, Figure 3 and Figure 4).

### 3.4. Multivariate Statistical Analyses

**Figure 6:**
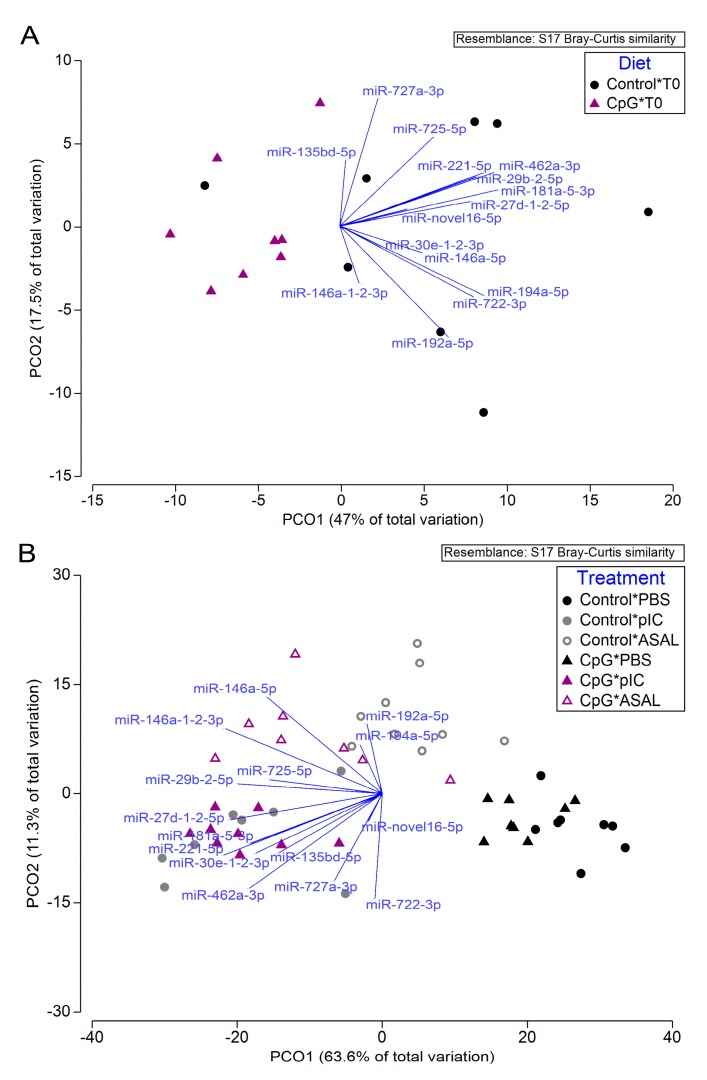
Principal coordinate analyses (PCoA) of qPCR analyzed miRNA genes (RQ values) in pre-injection samples (i.e., T0 samples; **A**) and 24 h post-injection head kidney samples (**B**).

For the pre-injection samples, the PCoA was able to segregate the two dietary groups (Figure 6A). miR-146a-5p, miR-27d-1-2-5p, and miR-181a-5-3p had the greatest influence on PCO1, which accounted for 47% of the variation among samples. PCO2 only explained 17.5% of the variability and was most strongly influenced by miR-727a-3p, miR-135bd-5p, and miR-146a-1-2-3p. For the post-injection groups, the PCoA was able to segregate different injection treatments and dietary groups within PBS- or ASAL-injected groups (Figure 6B). The top three miRNAs that influenced the PCO1 were miR-725-5p, miR-29b-2-5p, and miR-27d-1-2-5p, while PCO2 was mostly influenced by miR-192a-5p, miR-194a-5p, and miR-722-3p. PCO1 and PCO2 accounted for 63.6% and 11.3% of the variation among post-injected groups, respectively. PERMANOVA was conducted in order to quantify the differences among samples from fish fed different diets before and after stimulations. The results showed that the comparisons between diets within pre-injection and two of the post-injected groups (i.e., PBS and ASAL) were highly significant based on the expression of the 15 qPCR analyzed miRNAs (Table 5). SIMPER was conducted to explore the major drivers that differentiated dietary treatments. The comparison of miRNA expression between fish fed control and CpG diets within the ASAL treatment group was the most dissimilar (average dissimilarity = 26.81%), with 7 miRNAs (e.g., miR-146a-1-2-3p, miR-192a-5p, miR-221-5p, miR-29b-2-5p) as the top 70% contributing variables to this dissimilarity (Table 5). In the pre-injection and PBS-treated groups, the dissimilarities between diets were 17.52% and 19.0%, respectively. miR-194a-5p, miR-727a-3p, miR-725-5p, miR-722-3p, miR-192a-5p, and miR-181a-5-3p were the common contributing variables to both dissimilarities (Table 5).

## 4. Discussion

### 4.1. Deep Sequencing and Identification of Differentially Expressed miRNAs

In the current study, small RNA deep sequencing was used to discover miRNAs potentially involved in the antiviral and antibacterial immune responses in Atlantic salmon. The DESeq2 analyses of the head kidney from fish fed the control diet identified 12 and 18 miRNAs differentially expressed in pIC and ASAL groups, respectively, compared to PBS controls. It is well established that PAMPs can be detected by specific PRRs on or within the host cells, triggering immune responses [12]. In fish, pIC and ASAL have been used as models to study differentially expressed mRNAs associated with antiviral responses [5,13,14,15] and antibacterial responses [16,17], respectively. Similarly, various PAMPs (e.g., lipopolysaccharide, peptidoglycan, pIC) have been shown to modulate immune–relevant miRNA expression in teleosts [21,47,48,49]. Here we demonstrated that pIC and ASAL can be used to stimulate the immune responses that lead to changes in expression of many miRNAs known to be involved in host responses to infection.

The predicted target gene analyses of pIC- and/or ASAL-responsive miRNAs revealed that each of the miRNAs could regulate from 2 to 21 genes that have immune-relevant functions (Supplementary Tables S6 and S7). Some of these predicted target genes were mapped to KEGG pathways (e.g., NOD-like receptor signaling pathway, Toll-like receptor signaling pathway, RIG-I-like receptor signaling pathway or cytokine–cytokine receptor interaction pathway; see Table 4) that are important to host immune responses to viral and/or bacterial infection. This further confirms that the pIC- and/or ASAL-responsive miRNAs identified herein are relevant to the host-pathogen immune response.

The DESeq2 analyses of deep sequencing results were performed with a relatively small number of biological replicates (*n* = 3); therefore, there was a risk of false positives as well as false negatives. To avoid false positives, fifteen miRNAs identified as differentially expressed by DESeq2 were selected for qPCR confirmation using larger numbers of biological replicates (*n* = 8–9) than were included in the sequencing study. Among deep sequencing-identified pIC-responsive miRNAs subjected to qPCR analyses, only 1 miRNA (out of 8) did not confirm the DESeq2 results. For deep sequencing-identified ASAL-responsive miRNAs, 5 out of 10 were confirmed (i.e., statistically significant) by qPCR analyses. The library preparations of all samples involved in this study utilized identical chemistry and yielded similar percentages of high-quality reads. Therefore, the relative lower validation level of ASAL-responsive miRNA markers is unlikely to have been caused by the chemistry and/or sequencing-related factors. Rather, it is likely attributed to the difference in the sample sizes between sequencing and qPCR (i.e., 3 biological replicates in sequencing vs. 8–9 biological replicates in qPCR). In order to decrease the likelihood of false negatives, our future miRNA deep sequencing studies could employ higher numbers of biological replicates. For the remainder of the discussion, we will focus on the qPCR-confirmed miRNAs that were associated with antiviral and antibacterial immune responses.

### 4.2. miRNAs Associated with Both Antiviral and Antibacterial Immune Responses in Atlantic Salmon Head Kidney

The qPCR results showed that 5 miRNAs (miR-27d-1-2-5p, miR-29b-2-5p, miR-146a-5p, miR-146a-1-2-3p, and miR-221-5p) were significantly up-regulated by both pIC and ASAL stimulations when compared with the PBS-injection controls. The expression of miR-27d-1-2-5p in Atlantic salmon fry was recently shown to be decreased in fish challenged with IPNV at both 7 and 20 days post-infections [50]. In mice, miR-27 was reported to be down-regulated in multiple mouse cell lines and primary macrophages by cytomegalovirus (i.e., a DNA virus) infection; however, upon overexpression it exerted an antiviral function [51]. Compared with the pIC-induced miR-27d-1-2-5p in the present study, the opposite regulation of miR-27 by cytomegalovirus in mouse cells may be influenced by the virus–host interaction and/or difference in viral or PAMP nucleic acid (DNA virus vs. RNA pIC). In Asian seabass (*Lates calcarifer*), the up-regulation of miR-27 in the spleen was associated with LPS-induced inflammatory immune response [52]. In agreement with our findings, miR-29b was shown to be up-regulated in zebrafish embryos infected with *Salmonella typhimurium*, and in adult zebrafish infected with *Mycobacterium marinum* [53]. In contrast, the expression of miR-29 in Nile tilapia (*Oreochromis niloticus*) was down-regulated in fish challenged with *Streptococcus agalactiae* at early infection stages (i.e., 72 h) [54]. Interestingly, in mice, miR-29 was shown to suppress the interferon-gamma (IFN-γ) production by targeting IFN-γ mRNA directly, and miR-29 knockdown mice initiated more potent innate and type 1 helper T cell adaptive responses to intracellular bacterial infection [55]. Recently miR-29b-2-5p was also shown to respond to IPNV challenge in Atlantic salmon [50]. However, the functions of miR-27d-1-2-5p and miR-29b-2-5p in the antiviral and antibacterial responses in teleost fish including Atlantic salmon are yet to be determined.

In mammals, miR-146 was shown to regulate inflammatory responses following TLR-dependent pathogen recognition [56]. A study in human monocytes revealed that miR-146 attenuated TLR and cytokine signaling via a negative feedback loop involving the suppression of IL-1 receptor-associated kinase 1 and TNF receptor-associated factor 6 [57]. In fish, the expression of miR-146 increased in olive flounder (*Paralichthys olivaceus*) infected with VHSV [58], in Atlantic salmon infected with SAV [19], and in half-smooth tongue sole (*Cynoglossus semilaevis*) infected with *Vibrio anguillarum* [59]. In humans, studies have revealed that the expression of miR-221 is up-regulated in several types of cancers and was related to cancer cell proliferation [60,61,62]. Yan et al. [63] evaluated the expression of miR-221 in half-smooth tongue sole after challenge with *V. anguillarum*, and a head kidney cell line stimulated with different PAMPs. Similar to our study, the expression of miR-221 was increased in the liver and spleen of the infected sole, and the in vitro study indicated that both LPS and pIC up-regulated the expression of miR-221 at 6 h post-stimulation [63]. The expression of miR-221-5p was also evaluated in olive flounder in response to VHSV infection, showing increased transcription 24 h post-injection [58]. In olive flounder, miR-221-5p was predicted to target important immune genes (i.e., *cd18* and *irf5*) [58]. Recently, it was also shown to respond to IPNV infection in Atlantic salmon [50]. Taken together with our current results, it appears that miR-146 and miR-221 are involved in both antiviral and antibacterial immune responses and may play critical immune regulatory roles in Atlantic salmon.

### 4.3. miRNAs Only Associated with Antiviral Immune Response in Atlantic Salmon Head Kidney

Of the 4 miRNAs identified by deep sequencing as being responsive only to pIC, 3 (i.e., miR-30e-1-2-3p, miR-181a-5-3p, and miR-462a-3p) were qPCR confirmed as being significantly up-regulated in fish fed the control diet. Although there have been no studies to date reporting the association of miR-30e with antiviral immune response in Atlantic salmon, a previous study in Atlantic salmon infected with the intracellular bacterium *P. salmonis* did show increased miR-30e expression in both spleen and head kidney [23]. miR-181 and miR-462 were classified as evolutionarily conserved miRNAs associated with immune response in teleosts following viral challenges [18]. The expression of miR-181a was increased in response to red-spotted grouper nervous necrosis virus in a head kidney cell line of half-smooth tongue sole [49]. Similarly, Andreassen et al. [19] showed that SAV infection in Atlantic salmon caused the up-regulation of miR-181c-5p. The predicted target mRNAs of miR-181c-5p in Atlantic salmon included a number of immune-relevant genes such as neutrophil cytosolic factor 1 (*ncf1*), *irf3*, and inhibitor of nuclear factor kappa-B kinase subunit alpha-like (*ikbka*; alias: *chuk*) [19]. In mammals, it is suggested that the miR-181 family plays a central role in vascular inflammation by controlling critical signaling pathways (e.g., NF-κB signaling) and targets relevant to immune cell activation and homeostasis [64]. Similar to our findings, miR-462 was shown to be up-regulated in the spleen of flounder challenged with megalocytivirus (a DNA virus) infection [65], RTL-W1 (i.e., rainbow trout liver cell line) and Atlantic cod macrophages stimulated with pIC [21,66], and Atlantic salmon challenged with SAV [19]. Atlantic salmon miR-462a-3p was predicted to target macrosialin/CD68, a SAV-responsive gene [19]. Collectively, these results support the hypothesis that miR-30e-1-2-3p, miR-181a-5-3p, and miR-462a-3p have immune-related functions and possibly play important roles in the antiviral immune response in Atlantic salmon.

### 4.4. miRNAs Only Associated with Antibacterial Immune Response in Atlantic Salmon Head Kidney

Among the 6 miRNAs identified by deep sequencing as being responsive only to ASAL, miR-727a-3p was qPCR confirmed as being significantly down-regulated compared with PBS control in fish fed the control diet. Similarly, the expression of miR-727-3p was reduced in the LPS-stimulated blunt snout bream (*Megalobrama amblycephala*) [67]. It should be noted that miR-727 is likely a teleost-specific miRNA [68], and the role of miR-727a-3p in the antibacterial immune response in fish is yet to be determined.

Taken together, it seems that the 9 qPCR-confirmed miRNAs identified as pIC- and/or ASAL-responsive in our current study have some conserved immune-related functions and may play important regulatory roles in the antiviral and/or antibacterial immune responses in Atlantic salmon.

### 4.5. CpG Supplement Modulated the Expression of Immune-Relevant miRNAs in ASAL-Treated Atlantic Salmon

Unmethylated DNAs containing CpG motifs are PAMPs that are commonly used as immunostimulants in fish [28,29,30,31,32,33,69]. The immune response induced by CpG is mediated through TLR9, a PRR present on the cell surface or within endosomal compartments of immune cells [29]. Three different classes of CpG ODNs (A-, B-, and C-classes) have been characterized based on the backbone structure and sequence composition [28,69]. In humans, A-class CpG ODNs are known to activate type I IFN response, while B-class CpG ODNs (e.g., CpG ODN 1668, used in the current study) are more potent in B cell stimulation [70]. Similarly, Strandskog et al. [71] showed that A- and C-class CpG-ODNs induced strong IFN α/β activity, while B- and C-class CpG ODNs stimulated proliferation of leukocytes in Atlantic salmon. However, the impact of CpG as a functional feed ingredient on the modulation of immune-relevant miRNA expression in fish including Atlantic salmon had not been explored prior to our study.

To fully investigate the immune-modulating property of the CpG-containing diet in Atlantic salmon, we analyzed 15 DESeq2-identified miRNAs associated with immune response on the pre- and post-PAMP stimulation head kidney samples. The current qPCR analyses of candidate antiviral and antibacterial miRNAs showed that CpG supplementation generally suppressed basal expression of many miRNAs studied (i.e., in pre-injection samples). This may lead to the higher basal expression of genes targeted by these miRNAs. In post-injection groups (i.e., PBS and ASAL), however, many of these miRNAs were up-regulated in fish fed the CpG diet compared with fish fed the control diet. As shown by multivariate statistical analyses, dietary CpG had the most significant impact on the miRNA expression in the ASAL treated fish compared with other injection treatments, while the overall dietary CpG impact on candidate miRNAs expression in the pIC-injected fish was not significant (p (perm) = 0.4461). Seven miRNAs (e.g., miR-146a-1-2-3p, miR-192a-5p, miR-221-5p, and miR-29b-2-5p) were the most significant contributing variables to the dissimilarity between the ASAL-treated fish. As discussed above, many of these miRNAs, as shown in previous studies, are involved in immune responses of teleost fish.

Studies involving CpG administration via IP-injection showed that CpG ODN 1668 enhanced the immune responses of Pacific red snapper against *Vibrio parahaemolitycus* exposure and rock bream against iridovirus (a DNA virus) infection [31,32]. In contrast, studies in olive flounder revealed that CpG ODN 1668 conferred no protection against VHSV challenge and did not modulate the expression of well-known antiviral genes (i.e., *mx* and *isg15*), but elicited strong protection and immune response in fish challenged with a unicellular marine eukaryotic parasite *Miamiensis avidus* [69]. In our study, dietary CpG seemed to have no impact on the expression of candidate miRNAs in pIC-treated individuals, while inducing several miRNAs in the PBS- and ASAL-treated fish. This suggests that CpG ODN 1668 may modulate ASAL-stimulated antibacterial immune response rather than the pIC stimulated antiviral immune response based on candidate miRNA expression profiles. The selection of a CpG ODN appropriate to the characteristics of a specific pathogen (e.g., bacteria, virus or parasite) may be key to designing diets to improve defense against that pathogen. Finally, although the physiological and health-related consequences of the observed miRNA expression changes caused by the CpG diet remain unclear and require further study, it seems that selections of these miRNAs are suitable as immune-system associated biomarkers.

## 5. Conclusions

The present study identified and qPCR confirmed 9 miRNA biomarkers of Atlantic salmon response to pIC and/or ASAL immune stimulations. Many of the miRNAs identified herein are involved in immune responses, as shown in many similar teleost immune/pathogen challenge studies (discussed above). Regarding the immune-modulating properties of CpG diet on Atlantic salmon, we applied candidate miRNA biomarkers associated with immune response (identified in the current study) and evaluated the expression changes in pre- and post-stimulation individuals. CpG ODN 1668-containing diet may be useful in modulating the ASAL-triggered antibacterial immune response but not the pIC-triggered antiviral immune response of Atlantic salmon. Since the current study utilized pIC and ASAL rather than live pathogens, in the future it would be interesting to determine if dietary immunostimulant CpG ODN 1668 could have a protective effect in live bacterial pathogen challenges in Atlantic salmon. Finally, we anticipate that the molecular biomarkers identified herein will also be useful in the future development of functional feeds involving immunostimulants.

## Figures and Tables

**Table 1:** Overview of the deep sequencing results from the head kidney of fish fed the control diet.

Sample ID ^1^	Total Number of Raw Reads ^2^	Trimmed and Filtered Reads ^3^	Reads Mapped to miRNAs (%) ^4^	Accession Number ^5^
1-PBS-T30-2	9,609,300	4,800,941	89.1	SRR9709006
2-PBS-T30-3	15,384,722	9,966,411	90.4	SRR9709007
3-PBS-T33-3	12,674,361	8,124,580	89.9	SRR9709008
4-ASAL-T33-1	11,715,675	7,190,332	89.4	SRR9709009
5-ASAL-T30-4	14,028,188	9,134,360	89.7	SRR9709002
6-ASAL-T33-3	34,491,682	8,654,401	91.3	SRR9709003
7-pIC-T33-2	18,600,224	8,519,434	80.6	SRR9709004
8-pIC-T30-3	13,957,705	9,864,824	93.4	SRR9709005
9-pIC-T33-3	16,877,729	5,453,442	68.2	SRR9709010

^1^ The *mirVana*-prepared total RNAs from three of each PBS-, ASAL-, and pIC-injected individuals fed the control diet were selected for miRNA sequencing analyses. ^2^ The total number of reads in raw fastq file for each sample. ^3^ Total number of reads after removing adapters and filtering reads by size (18–25 nt). ^4^ Percent of trimmed and filtered reads mapped to the reference miRNAome (i.e., all known mature miRNAs of Atlantic salmon) [22]. ^5^ The accession number of sequencing results for each sample submitted to the NCBI Sequence Read Archive (SRA) database (http://www.ncbi.nlm.nih.gov/sra). All data were deposited under the BioProject PRJNA555179.

**Table 2:** pIC-responsive miRNAs in the head kidney of fish fed the control diet identified by DESeq2 (*n* = 3) and confirmed by qPCR (*n* = 8–9).

miRNAs ^1^	Base Mean ^2^	Fold-Change ^3^	Adjusted *p*-Values ^4^	qPCR Fold-Change ^5^
*Up-regulated by pIC*				
miR-27d-1-5p ^6^	868.11	2.17	0.049	2.69
miR-27d-2-5p ^6^	861.10	2.17	0.049	2.69
miR-30e-1-2-3p	404.73	2.16	0.049	2.57
miR-135bd-5p	52.80	14.03	0.036	1.54
**miR-146a-5p**	152066.51	1.71	0.028	1.62
**miR-146a-1-2-3p**	6322.03	7.94	3.85 × 10^-4^	7.89
miR-181a-5-3p	11080.58	1.59	0.051	1.97
**miR-221-5p**	221.52	4.44	0.036	7.55
miR-462a-3p	1554.09	2.69	0.098	5.72
miR-462b-3p	941.82	5.46	8.0 × 10^-12^	N/A
miR-8159-5p	97.72	9.92	0.055	N/A
*Down-regulated by pIC*				
miR-106a-3p	960.11	−1.93	0.036	N/A

^1^ miRNAs with bold font are differentially expressed in both pIC and ASAL groups when compared to the PBS-injected control group. The mature sequences and miRBase identities of each miRNA are given in Supplemental Table S4. ^2^ The mean of normalized read counts for all of the samples included in the comparison. ^3^ Fold-change (pIC/PBS) was converted from the log_2_ fold-change (given by DESeq2 analyses). For the down-regulated miRNA, the fold-change value was inverted (−1/fold-change). ^4^ Adjusted *p*-values as determined by DESeq2 analyses. ^5^ For qPCR fold-change calculation (fish fed the control diet), overall fold up-regulation was calculated as 2^A−B^ as in Xue et al. [46], where A was the mean of log_2_ RQ from the pIC group, and B was the mean of log_2_ RQ from PBS group. Underlined qPCR fold-change values indicate statistical significance (*p* < 0.05). N/A = not applicable. ^6^ miR-27d-2-5p and miR-27d-1-5p are identical except the length difference (24 vs. 23 nt). Therefore, the qPCR assay was generic to both miRNAs.

**Table 3:** ASAL-responsive miRNAs in the head kidney of fish fed the control diet identified by DESeq2 (*n* = 3) and confirmed by qPCR (*n* = 8–9).

miRNAs ^1^	Base Mean ^2^	Fold-Change ^3^	Adjusted *p*-Values ^4^	qPCR Fold-Change ^5^
*Up-regulated by ASAL*				
miR-21a-1-3p	267.99	1.52	0.099	N/A
miR-29b-2-5p	516.80	1.55	0.091	1.47
**miR-146a-5p**	202688.90	2.01	8.9 × 10^−6^	1.89
**miR-146a-1-2-3p**	8503.53	9.06	9.06 × 10^−37^	5.87
miR-146a-3-3p	12146.14	3.43	2.62 × 10^−11^	N/A
miR-146d-1-3p	421.43	2.60	2.45 × 10^−4^	N/A
miR-183-1-3-3p	19.55	4.17	0.018	N/A
miR-183-2-3p	19.24	4.08	0.021	N/A
miR-192a-5p	391.09	6.68	0.016	1.64
miR-194a-5p	82.25	6.68	0.016	1.37
miR-200b-3p	275.30	8.00	0.009	N/A
**miR-221-5p**	141.35	1.92	0.018	2.23
miR-429ab-3p	11.34	8.40	0.071	N/A
miR-725-5p	26.55	17.15	2.27 × 10^−6^	1.55
miR-725-3p	163.89	4.63	6.75 × 10^−6^	N/A
miR-novel-16-5p	1066.67	2.01	1.81 × 10^−4^	1.09
*Down-regulated by ASAL*				
miR-722-3p	410.45	−2.08	0.021	−1.64
miR-727a-3p	886.63	−1.82	0.085	−1.94

^1^ miRNAs with bold font were differentially expressed in both pIC and ASAL groups when compared to the PBS-injected control group. The mature sequences and miRBase identities of each miRNA are given in Supplemental Table S4. ^2^ The mean of normalized read counts for all of the samples included in the comparison. ^3^ Fold-change (ASAL/PBS) was converted from the log_2_ fold-change (given by DESeq2 analyses). For down-regulated miRNAs, fold-change values were inverted (−1/fold-change). ^4^ Adjusted *p*-values as determined by DESeq2 analyses. ^5^ For qPCR fold-change calculation (fish fed the control diet), overall fold up-regulation was calculated as 2^A−B^ as in Xue et al. [46], where A was the mean of log_2_ RQ from ASAL group, and B was the mean of log_2_ RQ from PBS group. Underlined qPCR fold-change values indicate statistical significance (*p* < 0.05). N/A = not applicable.

**Table 4:** Kyoto Encyclopedia of Genes and Genomes (KEGG) pathways of immune-relevant predicted target genes of pIC- and/or ASAL-responsive miRNAs.

Pathway	Name	Assigned Target Genes of pIC-Responsive miRNAs ^1^	Assigned Target Genes of ASAL-Responsive miRNAs ^2^
sasa04621	NOD-like receptor signaling pathway	8	12
sasa04060	Cytokine-cytokine receptor interaction	7	13
sasa04217	Necroptosis	7	11
sasa04620	Toll-like receptor signaling pathway	6	10
sasa04210	Apoptosis	6	7
sasa04625	C-type lectin receptor signaling pathway	5	5
sasa04622	RIG-I-like receptor signaling pathway	4	7
sasa04514	Cell adhesion molecules (CAMs)	4	2

^1^ Complete list of immune-relevant predicted target genes of pIC-responsive miRNAs is given in Supplemental Table S6. ^2^ Complete list of immune-relevant predicted target genes of ASAL-responsive miRNAs is given in Supplemental Table S7.

**Table 5:** Permutational multivariate ANOVA (PERMANOVA) and similarity of percentages analysis (SIMPER) of analyzed transcripts (RQ values).

	Control vs. CpG ^3^			
	Pre-Injection	PBS	pIC	ASAL
*p* (perm) ^1^	0.0015	0.0041	0.4461	0.0015
Average dissimilarity (%) ^2^	17.52	19.00	-	26.81
Contributing variables (top 70%)	miR-722-3p	miR-194a-5p	-	miR-146a-1-2-3p
	miR-192a-5p	miR-725-5p	-	miR-192a-5p
	miR-462a-3p	miR-192a-5p	-	miR-221-5p
	miR-727a-3p	miR-722-3p	-	miR-725-5p
	miR-194a-5p	miR-27d-1-2-5p	-	miR-29b-2-5p
	miR-725-5p	miR-727a-3p	-	miR-194a-5p
	miR-181a-5-3p	miR-146a-1-2-3p	-	miR-27d-1-2-5p
	miR-135bd-5p	miR-novel-16-5p	-	-
	-	miR-181a-5-3p	-	-

^1^*p* (perm) is the statistical significance value obtained from PERMANOVA with 9999 permutations. ^2^ Average dissimilarity and contributing variables (top 70%) were obtained through SIMPER. ^3^ Dietary effects were evaluated within each injection treatment.

## Supplementary Materials

The following are available online at https://www.mdpi.com/2073-4409/8/12/1592/s1. Supplemental Table S1. qPCR analyses of known ASAL and pIC immune biomarker transcripts in the head kidney of fish fed the control diet. Supplemental Table S2. miRNA qPCR primers. Supplemental Table S3. CT values of normalizers (miR-25-3p and miR-17-5p) in all analyzed samples. Supplemental Table S4. Mature sequences and miRBase identities of pIC- and/or ASAL-responsive miRNAs (identified by DESeq2 analyses). Supplemental Table S5. Predicted target genes of pIC- and/or ASAL-responsive miRNAs from the DESeq2 analyses. Supplemental Table S6. Immune-relevant predicted target genes of pIC-responsive miRNAs from the DESeq2 analysis. Supplemental Table S7. Immune-relevant predicted target genes of ASAL-responsive miRNAs from the DESeq2 analysis.

## References

[1] Agaba M.K., Tocher D.R., Zheng X., Dickson C.A., Dick J.R., Teale A.J. (2005). Cloning and functional characterisation of polyunsaturated fatty acid elongases of marine and freshwater teleost fish. Comp. Biochem. Physiol. Part. B Biochem. Mol. Biol..

[2] Tocher D.R., Zheng X., Schlechtriem C., Hastings N., Dick J.R., Teale A.J. (2006). Highly unsaturated fatty acid synthesis in marine fish: Cloning, functional characterization, and nutritional regulation of fatty acyl Δ6 desaturase of Atlantic cod (*Gadus morhua* L.). Lipids.

[3] FAO (2018). The State of World Fisheries and Aquaculture 2018—Meeting the Sustainable Development Goal.

[4] Kiron V. (2012). Fish immune system and its nutritional modulation for preventive health care. Anim. Feed Sci. Technol..

[5] Caballero-Solares A., Hall J.R., Xue X., Eslamloo K., Taylor R.G., Parrish C.C., Rise M.L. (2017). The dietary replacement of marine ingredients by terrestrial animal and plant alternatives modulates the antiviral immune response of Atlantic salmon (*Salmo salar*). Fish Shellfish Immunol..

[6] Lang A.S., Rise M.L., Culley A.I., Steward G.F. (2009). RNA viruses in the sea. FEMS Microbiol. Rev..

[7] Rise M.L., Jones S.R.M., Brown G.D., von Schalburg K.R., Davidson W.S., Koop B.F. (2004). Microarray analyses identify molecular biomarkers of Atlantic salmon macrophage and hematopoietic kidney responses to *Piscirickettsia salmonis* infection. Physiol. Genom..

[8] Ewart K.V., Belanger J.C., Williams J., Karakach T., Penny S., Tsoi S.C., Richards R.C., Douglas S.E. (2005). Identification of genes differentially expressed in Atlantic salmon (*Salmo salar*) in response to infection by *Aeromonas salmonicida* using cDNA microarray technology. Dev. Comp. Immunol..

[9] Murray A.G., Munro L.A., Wallace I.S., Allan C.E.T., Peeler E.J., Thrush M.A. (2012). Epidemiology of *Renibacterium salmoninarum* in Scotland and the potential for compartmentalised management of salmon and trout farming areas. Aquaculture.

[10] Løvoll M., Wiik-Nielsen C.R., Tunsjø H.S., Colquhoun D., Lunder T., Sørum H., Grove S. (2009). Atlantic salmon bath challenged with *Moritella viscosa*–Pathogen invasion and host response. Fish Shellfish Immunol..

[11] Vallejos-Vidal E., Reyes-López F., Teles M., MacKenzie S. (2016). The response of fish to immunostimulant diets. Fish Shellfish Immunol..

[12] Secombes C.J., Wang T., Austin B. (2012). The innate and adaptive immune system of fish. Infectious Disease in Aquaculture.

[13] Robertsen B. (2006). The interferon system of teleost fish. Fish Shellfish Immunol..

[14] Akira S., Uematsu S., Takeuchi O. (2006). Pathogen recognition and innate immunity. Cell.

[15] Eslamloo K., Xue X., Booman M., Smith N.C., Rise M.L. (2016). Transcriptome profiling of the antiviral immune response in Atlantic cod macrophages. Dev. Comp. Immunol..

[16] Feng C.Y., Johnson S.C., Hori T.S., Rise M., Hall J.R., Gamperl A.K., Hubert S., Kimball J., Bowman S., Rise M.L. (2009). Identification and analysis of differentially expressed genes in immune tissues of Atlantic cod stimulated with formalin-killed, atypical *Aeromonas salmonicida*. Physiol. Genom..

[17] Hori T.S., Gamperl A.K., Nash G., Booman M., Barat A., Rise M.L. (2013). The impact of a moderate chronic temperature increase on spleen immune-relevant gene transcription depends on whether Atlantic cod (*Gadus morhua*) are stimulated with bacterial versus viral antigens. Genome.

[18] Andreassen R., Høyheim B. (2017). miRNAs associated with immune response in teleost fish. Dev. Comp. Immunol..

[19] Andreassen R., Woldemariam N.T., Egeland I.Ø., Agafonov O., Sindre H., Høyheim B. (2017). Identification of differentially expressed Atlantic salmon miRNAs responding to salmonid alphavirus (SAV) infection. BMC Genom..

[20] Herkenhoff M.E., Oliveira A.C., Nachtigall P.G., Costa J.M., Campos V.F., Hilsdorf A.W., Pinhal D. (2018). Fishing into the MicroRNA transcriptome. Front. Genet..

[21] Eslamloo K., Inkpen S.M., Rise M.L., Andreassen R. (2018). Discovery of microRNAs associated with the antiviral immune response of Atlantic cod macrophages. Mol. Immunol..

[22] Woldemariam N.T., Agafonov O., Høyheim B., Houston R.D., Taggart J.B., Andreassen R. (2019). Expanding the miRNA repertoire in Atlantic salmon; discovery of isomiRs and miRNAs highly expressed in different tissues and developmental stages. Cells.

[23] Valenzuela-Miranda D., Valenzuela-Muñoz V., Farlora R., Gallardo-Escárate C. (2017). MicroRNA-based transcriptomic responses of Atlantic salmon during infection by the intracellular bacterium *Piscirickettsia salmonis*. Dev. Comp. Immunol..

[24] Cao Y., Wang D., Li S., Zhao J., Xu L., Liu H., Lu T., Mou Z. (2019). A transcriptome analysis focusing on splenic immune-related mciroRNAs of rainbow trout upon *Aeromonas salmonicida* subsp. salmonicida infection. Fish Shellfish Immunol..

[25] Tacchi L., Bickerdike R., Douglas A., Secombes C.J., Martin S.A.M. (2011). Transcriptomic responses to functional feeds in Atlantic salmon (*Salmo salar*). Fish Shellfish Immunol..

[26] Martin S.A.M., Król E. (2017). Nutrigenomics and immune function in fish: New insights from omics technologies. Dev. Comp. Immunol..

[27] Covello J.M., Friend S.E., Purcell S.L., Burka J.F., Markham R.J.F., Donkin A.W., Groman D.B., Fast M.D. (2012). Effects of orally administered immunostimulants on inflammatory gene expression and sea lice (*Lepeophtheirus salmonis*) burdens on Atlantic salmon (*Salmo salar*). Aquaculture.

[28] Carrington A.C., Secombes C.J. (2006). A review of CpGs and their relevance to aquaculture. Vet. Immunol. Immunopathol..

[29] Cuesta A., Esteban M.A., Meseguer J. (2008). The expression profile of TLR9 mRNA and CpG ODNs immunostimulatory actions in the teleost gilthead seabream points to a major role of lymphocytes. Cell. Mol. Life Sci..

[30] Liu C.-s., Sun Y., Hu Y.-h., Sun L. (2010). Identification and analysis of a CpG motif that protects turbot (*Scophthalmus maximus*) against bacterial challenge and enhances vaccine-induced specific immunity. Vaccine.

[31] Cárdenas-Reyna T., Angulo C., Hori-Oshima S., Velázquez-Lizárraga E., Reyes-Becerril M. (2016). B-cell activating CpG ODN 1668 enhance the immune response of Pacific red snapper (*Lutjanus peru*) exposed to *Vibrio parahaemolitycus*. Dev. Comp. Immunol..

[32] Jung M.-H., Jung S.-J. (2017). CpG ODN 1668 induce innate and adaptive immune responses in rock bream (*Oplegnathus fasciatus*) against rock bream iridovirus (RBIV) infection. Fish Shellfish Immunol..

[33] Purcell S.L., Friend S.E., Covello J.M., Donkin A., Groman D.B., Poley J., Fast M.D. (2013). CpG inclusion in feed reduces sea lice, *Lepeophtheirus salmonis*, numbers following re-infection. J. Fish. Dis..

[34] Chen J., Li C., Huang R., Du F., Liao L., Zhu Z., Wang Y. (2012). Transcriptome analysis of head kidney in grass carp and discovery of immune-related genes. BMC Vet. Res..

[35] Press C.M., Evensen Ø. (1999). The morphology of the immune system in teleost fishes. Fish Shellfish Immunol..

[36] Caballero-Solares A., Xue X., Parrish C.C., Foroutani M.B., Taylor R.G., Rise M.L. (2018). Changes in the liver transcriptome of farmed Atlantic salmon (*Salmo salar*) fed experimental diets based on terrestrial alternatives to fish meal and fish oil. BMC Genom..

[37] Martin M. (2011). Cutadapt removes adapter sequences from high-throughput sequencing reads. EMBnet J..

[38] Dobin A., Davis C.A., Schlesinger F., Drenkow J., Zaleski C., Jha S., Batut P., Chaisson M., Gingeras T.R. (2013). STAR: Ultrafast universal RNA-seq aligner. Bioinformatics.

[39] Liao Y., Smyth G.K., Shi W. (2013). featureCounts: An efficient general purpose program for assigning sequence reads to genomic features. Bioinformatics.

[40] Love M.I., Huber W., Anders S. (2014). Moderated estimation of fold change and dispersion for RNA-seq data with DESeq2. Genome Biol..

[41] Rehmsmeier M., Steffen P., Höchsmann M., Giegerich R. (2004). Fast and effective prediction of microRNA/target duplexes. RNA.

[42] Pfaffl M. (2001). A new mathematical model for relative quantification in real-time RT-PCR. Nucleic Acids Res..

[43] Johansen I., Andreassen R. (2014). Validation of miRNA genes suitable as reference genes in qPCR analyses of miRNA gene expression in Atlantic salmon (*Salmo salar*). BMC Res. Notes.

[44] Hellemans J., Mortier G., De Paepe A., Speleman F., Vandesompele J. (2007). qBase relative quantification framework and software for management and automated analysis of real-time quantitative PCR data. Genome Biol..

[45] Booman M., Xu Q.H., Rise M.L. (2014). Evaluation of the impact of camelina oil-containing diets on the expression of genes involved in the innate anti-viral immune response in Atlantic cod (*Gadus morhua*). Fish Shellfish Immunol..

[46] Xue X., Hixson S.M., Hori T.S., Booman M., Parrish C.C., Anderson D.M., Rise M.L. (2015). Atlantic salmon (*Salmo salar*) liver transcriptome response to diets containing *Camelina sativa* products. Comp. Biochem. Physiol. Part D Genom. Proteom..

[47] Chu Q., Sun Y., Bi D., Cui J., Xu T. (2017). Up-regulated of miR-8159-5p and miR-217-5p by LPS stimulation negatively co-regulate TLR1 in miiuy croaker. Dev. Comp. Immunol..

[48] Chu Q., Sun Y., Cui J., Xu T. (2017). MicroRNA-3570 modulates the NF-κB pathway in teleost fish by targeting MyD88. J. Immunol..

[49] Gong G., Sha Z., Chen S., Li C., Yan H., Chen Y., Wang T. (2015). Expression profiling analysis of the microRNA response of *Cynoglossus semilaevis* to *Vibrio anguillarum* and other stimuli. Mar. Biotechnol..

[50] Woldemariam N.T., Agafonov O., Sindre H., Høyheim B., Houston R.D., Robledo D., Bron J.E., Andreassen R. (2019). miRNAs predicted to regulate host anti-viral gene pathways in IPNV-challenged Atlantic salmon are affected by viral load, and associated with the major IPN resistance QTL genotypes in late infection. Front. Immunol..

[51] Buck A.H., Perot J., Chisholm M.A., Kumar D.S., Tuddenham L., Cognat V., Marcinowski L., Dölken L., Pfeffer S. (2010). Post-transcriptional regulation of miR-27 in murine cytomegalovirus infection. RNA.

[52] Xia J.H., He X.P., Bai Z.Y., Yue G.H. (2011). Identification and characterization of 63 MicroRNAs in the Asian seabass *Lates calcarifer*. PLoS ONE.

[53] Ordas A., Kanwal Z., Lindenberg V., Rougeot J., Mink M., Spaink H.P., Meijer A.H. (2013). MicroRNA-146 function in the innate immune transcriptome response of zebrafish embryos to *Salmonella typhimurium* infection. BMC Genom..

[54] Wang B., Gan Z., Cai S., Wang Z., Yu D., Lin Z., Lu Y., Wu Z., Jian J. (2016). Comprehensive identification and profiling of Nile tilapia (*Oreochromis niloticus*) microRNAs response to *Streptococcus agalactiae* infection through high-throughput sequencing. Fish Shellfish Immunol..

[55] Ma F., Xu S., Liu X., Zhang Q., Xu X., Liu M., Hua M., Li N., Yao H., Cao X. (2011). The microRNA miR-29 controls innate and adaptive immune responses to intracellular bacterial infection by targeting interferon-γ. Nat. Immunol..

[56] Pedersen I., David M. (2008). MicroRNAs in the immune response. Cytokine.

[57] Taganov K.D., Boldin M.P., Chang K.-J., Baltimore D. (2006). NF-κB-dependent induction of microRNA miR-146, an inhibitor targeted to signaling proteins of innate immune responses. Proc. Natl. Acad. Sci. USA.

[58] Najib A., Kim M.S., Choi S.H., Kang Y.J., Kim K.H. (2016). Changes in microRNAs expression profile of olive flounder (*Paralichthys olivaceus*) in response to viral hemorrhagic septicemia virus (VHSV) infection. Fish Shellfish Immunol..

[59] Sha Z., Gong G., Wang S., Lu Y., Wang L., Wang Q., Chen S. (2014). Identification and characterization of *Cynoglossus semilaevis* microRNA response to *Vibrio anguillarum* infection through high-throughput sequencing. Dev. Comp. Immunol..

[60] Galardi S., Mercatelli N., Farace M.G., Ciafre S.A. (2011). NF-kB and c-Jun induce the expression of the oncogenic miR-221 and miR-222 in prostate carcinoma and glioblastoma cells. Nucleic Acids Res..

[61] Lu X., Zhao P., Zhang C., Fu Z., Chen Y., Lu A., Liu N., You Y., Pu P., Kang C. (2009). Analysis of miR-221 and p27 expression in human gliomas. Mol. Med. Rep..

[62] Li J., Wang Y., Yu W., Chen J., Luo J. (2011). Expression of serum miR-221 in human hepatocellular carcinoma and its prognostic significance. Biochem. Biophys. Res. Commun..

[63] Yan H., Chen Y., Zhou S., Li C., Gong G., Chen X., Wang T., Chen S., Sha Z. (2016). Expression profile analysis of miR-221 and miR-222 in different tissues and head kidney cells of *Cynoglossus semilaevis*, following pathogen infection. Mar. Biotechnol..

[64] Sun X., Sit A., Feinberg M.W. (2014). Role of miR-181 family in regulating vascular inflammation and immunity. Trends Cardiovasc. Med..

[65] Zhang B.-c., Zhang J., Sun L. (2014). In-depth profiling and analysis of host and viral microRNAs in Japanese flounder (*Paralichthys olivaceus*) infected with megalocytivirus reveal involvement of microRNAs in host-virus interaction in teleost fish. BMC Genom..

[66] Schyth B.D., Bela-ong D.B., Jalali S.A.H., Kristensen L.B.J., Einer-Jensen K., Pedersen F.S., Lorenzen N. (2015). Two virus-induced MicroRNAs known only from teleost fishes are orthologues of MicroRNAs involved in cell cycle control in humans. PLoS ONE.

[67] Yuhong J., Leilei T., Fuyun Z., Hongyang J., Xiaowen L., Liying Y., Lei Z., Jingrong M., Jinpeng Y. (2016). Identification and characterization of immune-related microRNAs in blunt snout bream, *Megalobrama amblycephala*. Fish Shellfish Immunol..

[68] Zhu Y.-P., Xue W., Wang J.-T., Wan Y.-M., Wang S.-L., Xu P., Zhang Y., Li J.-T., Sun X.-W. (2012). Identification of common carp (*Cyprinus carpio*) microRNAs and microRNA-related SNPs. BMC Genom..

[69] Kang Y.J., Kim K.H. (2012). Effect of CpG-ODNs belonging to different classes on resistance of olive flounder (*Paralichthys olivaceus*) against viral hemorrhagic septicemia virus (VHSV) and *Miamiensis avidus* (Ciliata; Scuticociliatia) infections. Aquaculture.

[70] Kerkmann M., Rothenfusser S., Hornung V., Towarowski A., Wagner M., Sarris A., Giese T., Endres S., Hartmann G. (2003). Activation with CpG-A and CpG-B oligonucleotides reveals two distinct regulatory pathways of type I IFN synthesis in human plasmacytoid dendritic cells. J. Immunol..

[71] Strandskog G., Ellingsen T., Jørgensen J.B. (2007). Characterization of three distinct CpG oligonucleotide classes which differ in ability to induce IFN α/β activity and cell proliferation in Atlantic salmon (*Salmo salar* L.) leukocytes. Dev. Comp. Immunol..

